# Gestational Diabetes and Thyroid Autoimmunity

**DOI:** 10.1155/2012/867415

**Published:** 2012-05-13

**Authors:** Ester Vitacolonna, Annunziata Lapolla, Barbara Di Nenno, Annalisa Passante, Ines Bucci, Cesidio Giuliani, Dominique Cerrone, Fabio Capani, Fabrizio Monaco, Giorgio Napolitano

**Affiliations:** ^1^Clinical Research Center (CRC), “Gabriele d'Annunzio” University Foundation, 66013 Chieti, Italy; ^2^“Leonardo da Vinci” Online University, Torrevecchia Teatina, 66013 Chieti, Italy; ^3^Chair of Metabolic Diseases, Department of Clinical and Surgical Sciences, University of Padua, 35122 Padua, Italy; ^4^Section of Endocrinology, Centre for Aging Sciences (CESI), “Gabriele d'Annunzio” University Foundation, 66013 Chieti, Italy; ^5^Section of Endocrinology, Department of Human Movement Sciences, “Gabriele d'Annunzio” University, 66013 Chieti, Italy; ^6^Section of Endocrinology, Department of Medicine and Sciences of Aging, “Gabriele d'Annunzio” University, 66013 Chieti, Italy

## Abstract

*Background*. About 10% of pregnancies are complicated by previously unknown impairment of glucose metabolism, which is defined as gestational diabetes. There are little data available on prevalence of thyroid disorders in patients affected by gestational diabetes, and about their postgestational thyroid function and autoimmunity. We therefore investigated pancreatic and thyroid autoimmunity in gestational diabetic patients and in women who had had a previous gestational diabetic pregnancy. *Methods*. We investigated 126 pregnant women at the time of a 100-g oral glucose tolerance test: 91 were classified as gestational diabetics, and 35 were negative (controls). We also studied 69 women who had delivered a baby 18–120 months prior to this investigation and who were classified at that time gestational diabetics (38 women) or normally pregnant (31 women; controls). *Results*. Our data show no differences for both thyroid function and prevalence of autoimmune disorders during pregnancy; however, a significant increase in thyroid autoimmunity was seen in women previously affected by gestational diabetes. This increased prevalence of thyroid autoimmunity was not associated with the development of impaired glucose metabolism after pregnancy. *Conclusions*. Our data suggest that maternal hyperglycemia is a risk factor for the development of thyroid autoimmunity, a conclusion that should now be confirmed in a larger cohort of patients.

## 1. Introduction

Gestational diabetes (GDM) is defined as any degree of carbohydrate intolerance that is first diagnosed during pregnancy [1]. The prevalence of GDM ranges between 1% and 14% [1–3], and it is most frequent in women aged ≥35 years in the second trimester of pregnancy. In a minority (<10%) of patients, the need for insulin therapy continues after delivery, and in these cases detection of anti-glutamic-acid-decarboxylase- (GAD-) 65 antibodies (GAD65-Ab) is common, and so GDM is considered the onset of type 1 diabetes [4–6]. Increased incidence of organ-specific autoimmunity towards endocrine cells other than *β*-cells has been described in type 1 diabetes patients [7], and it is believed to be caused by a genetic propensity to autoimmune disorders. In the majority of patients, however, GDM is believed to be caused by *β*-cell dysfunction that occurs on a background of chronic insulin resistance. Although in these patients GDM usually resolves after delivery, up to 70% of them develop overt type 2 diabetes mellitus within 10 years [4, 8].

Recent studies have reported increased incidence of thyroid autoimmunity in type 2 diabetes [9, 10], thus implying that diabetes can trigger the onset of thyroid autoimmunity. Few studies have, however, evaluated the prevalence of thyroid dysfunction and autoimmunity in women with GDM [11–13]. Moreover, little is known about thyroid autoimmunity in post-GDM patients, who usually return to normal glucose control after delivery. In our opinion, GDM offers a good opportunity to study if diabetes and hyperglycemia might predispose to thyroid autoimmunity.

In the present study, we therefore investigated (1) pancreatic and thyroid autoimmunity in GDM patients and (2) pancreatic and thyroid autoimmunity in women with previous GDM.

## 2. Patients and Methods

### 2.1. Patients

One-hundred-and-ninety-five women (aged 18 to 51) who were referred to the Diabetic Outpatient Clinic of the “Santo Spirito” Pescara Hospital were evaluated; none of these patients had a hyperglycemic disease before gestation. Initially, 126 consecutive pregnant (group A) women were evaluated at the 14th to 34th weeks of pregnancy at the time of a 100 g oral glucose tolerance test (OGTT), which was interpreted according to the O' Sullivan and Mahan criteria revised by Carpenter and Coustan [14–16]. According to the OGTT results, these patients were further subdivided into two groups.

- A1: 91 patients (mean age, 33 ± 10 years) with a positive OGTT, considered as women affected by GDM.
- A2: 35 women (mean age, 29 ± 11 years) with a negative OGTT, considered as normally pregnant women (controls).

The remaining 69 women (group B) had delivered a baby 18 months to 120 months before this study. During their pregnancy, they had been evaluated by an OGTT and were diagnosed accordingly as having been affected, or not, by GDM. For the purpose of this study, these patients were also further subdivided into two groups.

- B1: 38 patients (mean age, 40 ± 11 years) with a positive OGTT during their pregnancy, considered as women previously affected by GDM. At the time of the present study (18–120 months after delivery), they were evaluated again for thyroid function and glucose metabolism by a 75 g OGTT.
- B2: 31 women (mean age, 40 ± 5.7 years) with a negative OGTT during their pregnancy who did not develop diabetes after delivery, considered as previously normal pregnancy controls; the time elapsed since their delivery was 18 months to 96 months. They were enrolled as mothers whose children were attending the family care clinic of two pediatricians operating in the Pescara area.

Women with a positive history for thyroid diseases and who had taken any drug known to interfere with thyroid function or the immune system were excluded from the study. None of the subjects enrolled in the study were evaluated twice; therefore, none of the patients in group A were also included in group B. All women with elevated TSH levels in groups A1 and A2 were treated with L-thyroxine; for women of group B1 we individually evaluated the option of treatment. Informed consent was obtained from all of the participants in the study.

### 2.2. Methods

Commercially available kits were used to determine the levels of free thyroxine (FT4) (immunofluorometric assay; normal range 0.76–1.42 ng/dL; Perkin Elmer Italia spa, Monza, Italy), thyrotropin (TSH) (immunofluorometric assay TSH ultra; normal range 0.4–4.2 *μ*IU/L; Perkin Elmer Italia spa, Monza, Italy), antiperoxidase antibodies (TPO) (TPO-Ab radioimmunoassay kit; normal value <15 IU/mL; Becton, Dickinson and Co, Franklin Lakes, NJ, USA), antithyroglobulin antibodies (Tg) (Tg-Ab Elisa Kit; normal value <100 IU/mL; Alpco Diagnostics, Salem, NH, USA), and antiglutamic acid decarboxylase-65 antibodies (GAD65-Ab radioimmunoassay; normal value <1 IU/mL; Adaltis Italia spa, Casalecchio di Reno, Italy). 

Stimulating TSH receptor antibodies (TSHr-Abs) were calculated using a biological assay, as previously described [17]. Briefly, Chinese hamster ovary (CHO) cells were subjected to a two-step double stable transfection: in the first step, the cells were transfected with a CRE-luc construct, which makes the cells particularly sensitive to changes in cAMP levels. In the second step, the cells were transfected with wild-type human TSHr. These cells were maintained in Ham's F12 nonessential amino acids supplemented with 10% fetal calf serum and penicillin/streptomycin (1 U/mL/1 mg/mL, resp.) for 24 hours, which was then replaced with starvation media (Hank's balanced salt solution; no fetal calf serum) for another 24 hours. All of the cells were maintained at 37°C, in 5% CO_2_ and at 95% relative humidity. Cut-off of normal values was determined in the following manner: the mean of ≥5 samples from normal subjects was calculated. The standard deviation between these normal samples was determined, and this value was multiplied by two and added to the calculated mean. The cut-off value obtained in this way was arbitrarily considered equal to 1 unit (AU). The luciferase activity was determined using the Bright-Glow reagent, by measurement of the light output using a single-tube luminometer. Interassay and intra-assay variability were <5%. 

Student' *T* tests were used with unpaired data, and Fisher's exact tests and *χ* ^2^ tests were performed.

## 3. Results

The presence of pancreatic autoimmunity in the cohort of patients was evaluated by determination of GAD65-Ab levels. We observed that 3 (3.3%) patients were positive for GAD65-Ab in group A1, 2 of whom had low GAD-65 titers (<2 IU/mL), while the third had a GAD-65 titer of 33 IU/mL. All of the patients in group A2 were negative for GAD65-Ab. In the group of previously gestational women (B1), 2 (5.3%) patients had GAD65-Ab-positive values, 1 of whom had low level positivity (<2 IU/mL), and the second, who had a GAD65-Ab value of 16 IU/mL, was diagnosed as type 1 diabetic and is on insulin treatment. None of the B2 group (control group) showed positive GAD65-Ab values (Table 1). 

**Figure 1:**
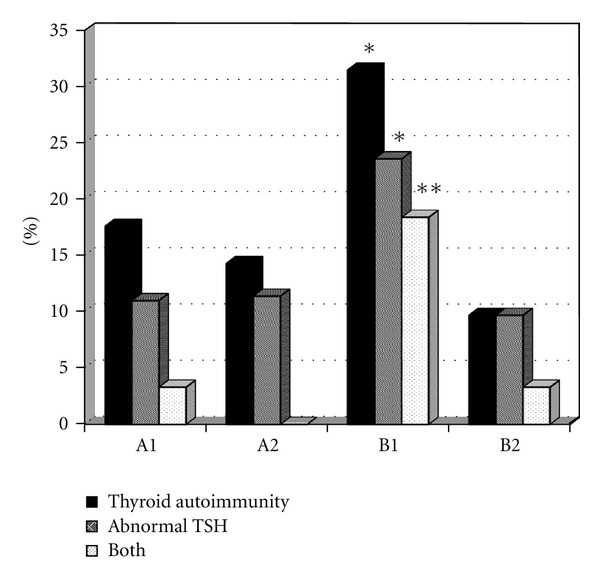
Patients (as percentages within each single group) affected by thyroid autoimmunity (TPO-Ab, Tg-Ab, and TSHr-Ab positivity), thyroid dysfunction (TSH < 0.4 mIU/L or >4.2 mIU/L) or the combination. Group A1: GDM pregnant women; group A2: non-GDM pregnant women; group B1: post-GDM women; group B2: healthy mothers. *P < 0.05 versus groups A1 and B2; **P < 0.05 versus group B2 and *P* < 0.001 versus group A1.

The mean TSH values were not significantly different between the four groups (1.46 ± 1.02 versus 1.90 ± 1.4 mIU/L in groups A1 and A2, resp., and 2.45 ± 4.32 versus 1.44 ± 0.92 mIU/L in groups B1 and B2, resp.) (Table 1). No significant differences were seen between the four groups either considering these mean TSH values or separately evaluating the TSH values as below and above the normal range. However, if we consider the overall incidence of abnormal TSH values, a significantly higher incidence (*P* < 0.05) was seen for group B1 versus both groups A1 and B2 (Figure 1). 

The FT4 levels were not significantly different when comparing either gestational versus normal pregnancies (0.82 ± 0.13 versus 0.83 ± 0.09 ng/dL for groups A1 and A2, resp.) or postgestational versus controls (0.94 ± 0.21 versus 0.92 ± 0.17 ng/dL for groups B1 and B2, resp.) (Table 1). Our data confirm the significantly lower FT4 values in pregnancy (0.82 ± 0.13 versus 0.93 ± 0.19 ng/dL, resp.; *P* < 0.001); however, FT4 was within the normal range in all of the pregnant women if the values are modified according to pregnancy [18]. 

Anti-TPO-Abs were detected in 16 (17.6%) patients in group A1, 5 (14.3%) in group A2, 10 (26.3%) in group B1, and 3 (9.7%) in group B2. Anti-Tg-Abs were detected in 6 (6.6%) patients in group A1, 1 (2.8%) in group A2, 6 (15.8%) in group B1, and 1 (3.2%) in group B2. Moreover, 1 patient in group B1 (of previously gestational women) had positive stimulating TSHr-Ab, with a suppressed TSH (<0.01 *μ*IU/mL) and negative TPO-Ab. Therefore, the overall incidence of thyroid autoimmunity (12/38, 31.6%) in group B1 was significantly (*P* < 0.05) greater than in groups B2 and A1 (Figure 1). No other subjects were positive for stimulating TSHr-Ab. Only 1 patient (in group A1) had positivity for both GAD65-Ab and TPO-Ab. 

When considering the coincident presence of thyroid autoimmunity and abnormal TSH values, it is interesting to note that the combination of both was observed in 3/91 (3.3%) patients in group A1, 0/35 (0.0%) in group A2, 7/38 (18.4%) in group B1, and 1/31 (3.3%) in group B2. The association of abnormal TSH and TPO-Ab positivity was significantly greater in group B1 versus both groups B2 (*P* < 0.05) and A1 (*P* < 0.001) (Table 1 and Figure 1). 

The possibility that thyroid Ab positivity is associated with the onset of permanently impaired glucose metabolism in the previously gestational women (Table 2) was also considered. Here, 18/38 (47.4%) previously gestational women showed hyperglycemic disease at the follow-up; these patients were widely distributed between thyroid autoimmune and nonautoimmune patients. Indeed, 7/12 (58.3%) women with thyroid Ab (TPO-Ab and TSHr-Ab) positivity showed hyperglycemia, while 11/26 (42.3%) with negative thyroid Abs had impaired glucose metabolism; no statistically significant differences were detected between these two groups.

## 4. Discussion

The prevalence of pancreatic autoimmunity in GDM has been widely investigated [3, 5, 6, 19], and it has been shown to differ for racial and geographic reasons. In the present study, GAD65-Abs were detected in 3.3% of our population, a level that is in agreement with several previous reports [2, 3, 5, 6, 19]. In our study we have chosen to determine only anti-GAD, because GAD autoantibodies are markers with the highest diagnostic sensitivity in LADA, so they should be used to identify such patient [20]. 

Fewer studies have investigated the prevalence of thyroid autoimmunity during GDM: most of these did not show a significant increase [13, 21], although few reports [11, 22] showed a higher risk of thyroid autoimmunity in women with a family history of diabetes and thyroid diseases. The present study also shows no significant differences. 

The mean TSH value of the GDM patients was similar to that seen in normally pregnant women, and no differences were seen relating to the prevalence of abnormal TSH values between these two groups. At the same time, the FT4 levels were not significantly different. In summary, it can be concluded that no differences in thyroid function and autoimmunity were detected in the present study in GDM patients, as compared with normally pregnant women. 

To our knowledge, little data are available on the prevalence of thyroid autoimmunity in women with previous GDM. We found an increased frequency of thyroid antibodies in patients with previous GDM; indeed, 31.6% of our patients were positive for TPO-Ab, Tg-Abs or TSHr-Ab, as compared with 9.7% for women with previously normal pregnancies. We would also underline that in one patient, thyroid autoimmunity was only revealed by the TSHr-Ab assay. The presence of the TSHr-Ab as the only marker of thyroid autoimmunity has been described previously for a population of type 1 diabetes patients [23]; however, GAD65-Abs were negative in our patients, and there is no need at present for insulin therapy. The TSHr-Ab assay has therefore to be considered as a useful tool to reveal subclinical autoimmune hyperthyroidism whenever TSH is below the normal range, even if TPO-Abs are negative and there is no other sign of endocrine autoimmunity. 

An association of thyroid dysfunction and Ab positivity was detected more frequently in the previously gestational women (7/38 [18.4%] in group B1, and 1/31 [3.3%] in group B2). On the basis of our data, it can be speculated that an increase in thyroid autoimmunity occurs in post-GDM women and that this phenomenon is relevant enough to cause subclinical thyroid dysfunction. 

An association between autoimmune diabetes (type 1 and latent autoimmune diabetes) and other organ-specific autoimmune disorders has been widely described [7, 24]; however, in the present study, only 1 woman showed positivity for both pancreatic and thyroid autoimmunities. A common (pancreatic and thyroid) autoimmune trait appears therefore an unlikely explanation for the increased prevalence of thyroid autoimmunity in our post-GDM patients. Igawa et al. [25] suggested that the clinical association between chronic autoimmune thyroiditis and type 2 diabetes is related to a common antigen that is shared by pancreatic *β*-cells and thyroid follicular cells. More recently, we have shown that a 10 mM increase in glucose levels in cultured thyroid cells can upregulate major histocompatibility complex (MHC) class I expression [26]. We therefore hypothesized that this phenomenon causes the thyrocyte to become an antigen-presenting cell and possibly to overcome self-tolerance. It has indeed been shown that elevated levels of MHC molecules, which increase thyroid antigen presentation, can trigger thyroid autoimmunity in animal models [27, 28]. 

In accordance with this observation, we speculate that hyperglycemia at the time of pregnancy or immediately after delivery triggered the autoimmune disorder in our patients. If this is the case, GDM represents a unique chance to evaluate progression of thyroid autoimmunity from its onset. 

It has to be emphasized, however, that the present study and our speculative explanation should still be considered as a preliminary step. In our study the diagnosis of GDM was carried out by OGTT with 100 g of glucose, and as is known new diagnostic criteria suggested by IADPSG [29] and ADA have been recently introduced. We cannot say whether the conduct would have been the same with new criteria. In fact the new criteria for diagnosing GDM identified a group of women previously classifiable as normal according to the 4th International Workshop Conference criteria, but revealing metabolic characteristics and pregnancy outcomes resembling those of women who would have been considered to have gestational diabetes by the previous criteria [30]. 

 A major limit of this study is indeed the relatively small number of subjects evaluated. Therefore, observations on a larger population are needed to confirm our data. 

In this context, we also cannot rule out the possibility that progression towards hyperglycemic disease after delivery can further facilitate the onset of thyroid autoimmune disease; the small number of patients did not allow the reaching of statistical significance, even if a higher percentage of patients with impaired glucose metabolism showed thyroid Ab positivity (58.3% versus 42.3%). In a 20-year follow-up, Männistö and colleagues [31] showed that instead of thyroid antibodies presence of overt hypothyroidism poses risk of diabetes. On the other side, the presence of TPO-Ab is not predisposing to the development of GDM [32]. Indeed, in a large study that included more than 600 pregnant women, GDM was seen in 8.1% of the women with TPO-antibody positivity, as compared to 6.8% without the TPO antibody, and this difference was not significant. 

In summary, the main and new finding from our study is the higher prevalence of thyroid autoimmunity in women who have had previous GDM; in the same group, thyroid dysfunction is also more prevalent. We speculate that gestational hyperglycemia can trigger thyroid autoimmunity.

##  Conflict of Interests

The authors declare that they have no competing financial interests.

## Figures and Tables

**Table 1:** Clinical characteristics and laboratory data of the four groups of women evaluated.

	Groups			
	A1 (*n* = 91)	A2 (*n* = 35)	B1 (*n* = 38)	B2 (*n* = 31)
Mean age (years)	33 ± 10	29 ± 11	40 ± 11	40 ± 6
Time from delivery (range, months)	—	—	18–120	18–96
BMI at OGTT	29.8 ± 4.0	28.1 ± 4.7	27.2 ± 2.2	—
BMI at follow-up	—	—	24.1 ± 2.8	23.5 ± 3.7
GAD65-Ab positivity [*n* (%)]	3 (3.3)	0	2 (5.3)	0
Mean FT4 (ng/dL)	0.82 ± 0.13	0.83 ± 0.09	0.93 ± 0.21	0.92 ± 0.17
Mean TSH (mIU/L)	1.46 ± 1.02	1.90 ± 1.4	2.45 ± 4.32	1.44 ± 0.92
TSH < 0.4 mIU/L [*n* (%)]	8 (8.8)	2 (5.7)	5 (13.1)	2 (6.5)
TSH > 4.2 mIU/L [*n* (%)]	2 (2.2)	2 (5.7)	4 (10.5)	1 (3.2)
Overall abnormal TSH [*n* (%)]	10 (11.0)	4 (11.4)	9 (23.7)*	3 (9.7)
Thyroid Ab positivity [*n* (%)]	16 (17.6)	5 (14.4)	12 (31.6)*	3 (9.7)
Abnormal TSH + thyroid Ab positivity [*n* (%)]	3 (3.3)	0 (−)	7 (18.4)**	1 (3.2)

*P < 0.05 versus groups A1 and B2; **P < 0.05 versus groups B2 and *P* < 0.001 versus group A1. Where indicated, data are means ± SD.

**Table 2:** Glucose abnormalities and thyroid autoimmunity in post-GDM women (group B1).

Thyroid autoimmunity	Condition	Glucose metabolism [*n* (%)]
Thyroid Ab positive (*n* = 12)	Normal	5 (41.7)
	Type 1 diabetes mellitus	1 (8.3)
	Type 2 diabetes mellitus	2 (16.7)
	Impaired glucose tolerance	3 (25)
	Impaired fasting glucose	1 (8.3)

Thyroid Ab negative (*n* = 26)	Normal	15 (57.7)
	Type 1 diabetes mellitus	1 (3.8)
	Type 2 diabetes mellitus	2 (7.7)
	Impaired glucose tolerance	8 (30.8)
	Impaired fasting glucose	—

## References

[1] American Diabetes Association (2011). Diagnosis and classification of diabetes mellitus (Position Statement). *Diabetes Care*.

[2] King H (1998). Epidemiology of glucose intolerance and gestational-diabetes in women of childbearing age. *Diabetes Care*.

[3] Lapolla A, Dalfrà MG, Lencioni C, Di Cianni G (2004). Epidemiology of diabetes in pregnancy: a review of Italian data. *Diabetes, Nutrition and Metabolism*.

[4] Buchanan TA, Xiang AH (2005). Gestational diabetes mellitus. *Journal of Clinical Investigation*.

[5] Lapolla A, Fedele D, Pedini B (2002). Low frequency of autoantibodies to islet cell, glutamic acid decarboxylase, and second-islet antigen in patients with gestational diabetes mellitus: a follow-up study. *Annals of the New York Academy of Sciences*.

[6] Damm P, Kuhl C, Buschard K (1994). Prevalence and predictive value of islet cell antibodies and insulin autoantibodies in women with gestational diabetes. *Diabetic Medicine*.

[7] Barker JM (2006). Clinical review: type 1 diabetes-associated autoimmunity: natural history, genetic association and screening. *Journal of Clinical Endocrinology and Metabolism*.

[8] Bellamy L, Casas JP, Hingorani AD, Williams D (2009). Type 2 diabetes mellitus after gestational diabetes: a systematic review and meta-analysis. *The Lancet*.

[9] Chubb SA, Davis WA, Davis TM (2005). Interactions among thyroid function, insulin sensitivity and serum lipid concentration: the Freemantle diabetes study. *Journal of Clinical Endocrinology and Metabolism*.

[10] Chubb SAP, Davist WA, Inman Z, Davis TME (2005). Prevalence and progression of subclinical hypothyroidism in women with type 2 diabetes: the fremantle diabetes study. *Clinical Endocrinology*.

[11] Olivieri A, Valensise H, Magnani F (2000). High frequency of antithyroid autoantibodies in pregnant women at increased risk of gestational diabetes mellitus. *European Journal of Endocrinology*.

[12] Olivieri A, Pinna G, Lai A (2001). The Sardinian autoimmunity study 4. Thyroid and islet cell autoantibodies in Sardinian pregnant women at risk at delivery: a cross-sectional study. *Journal of Endocrinological Investigation*.

[13] Agarwal M, Dhatt G, Punnose J, Bishawi B, Zayed R (2006). Thyroid function abnormalities and antithyroid antibody prevalence in pregnant women at high risk for gestational diabetes mellitus. *Gynecological Endocrinology*.

[14] O’Sullivan JB, Mahan CM (1964). Criteria for oral glucose tolerance test in pregnancy diabetes. *Diabetes*.

[15] Carpenter MW, Coustan DR (1982). Criteria for screening tests for gestational diabetes. *American Journal of Obstetrics and Gynecology*.

[16] Metzger BE, Buchanan TA, Coustan DR (2007). Summary and recommendations of the Fifth International Workshop-Conference on Gestational Diabetes Mellitus. *Diabetes Care*.

[17] Tahara K, Ban T, Minegishi T, Kohn LD (1991). Immunoglobulins from Graves’ disease patients interact with different sites on TSH receptor/LH-CG receptor chimeras than either TSH or immunoglobulins from idiopathic myxedema patients. *Biochemical and Biophysical Research Communications*.

[18] Soldin OP, Tractenberg RE, Hollowell JG, Jonklaas J, Janicic N, Soldin SJ (2004). Trimester-specific changes in maternal thyroid hormone, thyrotropin, and thyroglobulin concentrations during gestation: trends and associations across trimesters in iodine sufficiency. *Thyroid*.

[19] Weng J, Ekelund M, Lehto M (2002). Screening for MODY mutations, GAD antibodies, and type 1 diabetes-associated HLA genotypes in women with gestational diabetes mellitus. *Diabetes Care*.

[20] Lapolla A, Dalfra MG, Fedele D (2009). Diabetes related autoimmunity in gestational diabetes mellitus: is it important?. *Nutrition, Metabolism and Cardiovascular Diseases*.

[21] Ortega-González C, Liao-Lo A, Ramírez-Peredo J, Cariño N, Lira J, Parra A (2000). Thyroid peroxidase antibodies in Mexican-born healthy pregnant women, in women with type 2 or gestational diabetes mellitus, and in their offspring. *Endocrine Practice*.

[22] Nakova VV, Krstevska B, Dimitrovski C, Simeonova S, Hadzi-Lega M, Serafimoski V (2010). Prevalence of thyroid dysfunction and autoimmunity in pregnant women with gestational diabetes and diabetes type 1. *Prilozi*.

[23] Unnikrishnan AG, Kumaravel V, Nair V (2006). TSH receptor antibodies in subjects with type 1 diabetes mellitus. *Annals of the New York Academy of Sciences*.

[24] Gambelunghe G, Forini F, Laureti S (2000). Increased risk for endocrine autoimmunity in Italian type 2 diabetic patients with GAD65 autoantibodies. *Clinical Endocrinology*.

[25] Igawa T, Nakabayashi H, Takeda R, Kurata Y (1996). A possible common cell surface autoantigen in islet *β*-cells and thyroid follicular cells in patients with non-insulin dependent diabetes mellitus and chronic thyroiditis. *Endocrine Journal*.

[26] Napolitano G, Bucci I, Giuliani C (2002). High glucose levels increase major histocompatibility complex class I gene expression in thyroid cells and amplify interferon-*γ* action. *Endocrinology*.

[27] Shimojo N, Kohno Y, Yamaguchi KI (1996). Induction of Graves-like disease in mice by immunization with fibroblasts transfected with the thyrotropin receptor and a class II molecule. *Proceedings of the National Academy of Sciences of the United States of America*.

[28] Verma S, Hutchings P, Guo J, McLachlan S, Rapoport B, Cooke A (2000). Role of MHC class I and CD8(+) cells in the evolution of iodine-induced thyroiditis in NOD-H2(h4) and NOD mice. *European Journal of Immunology*.

[29] International Association of Diabetes and Pregnancy Study Groups Consensus Panel (2010). Recommendations on the diagnosis and classification of hyperglycemia in pregnancy. *Diabetes Care*.

[30] Lapolla A, Dalfrà MG, Ragazzi E, de Cata AP, Fedele D (2011). New international association of the diabetes and pregnancy study groups (IADPSG) recommendations for diagnosing gestational diabetes compared with former criteria: a retrospective study on pregnancy outcome. *Diabetic Medicine*.

[31] Männistö T, Vääräsmäki M, Pouta A (2010). Thyroid dysfunction and autoantibodies during pregnancy as predictive factors of pregnancy complications and maternal morbidity in later life. *Journal of Clinical Endocrinology and Metabolism*.

[32] Montaner P, Juan L, Campos R, Gil L, Corcoy R (2008). Is thyroid autoimmunity associated with gestational diabetes mellitus?. *Metabolism*.

